# Modified CBT for social anxiety and social functioning in young adults with autism spectrum disorder

**DOI:** 10.1186/s13229-021-00418-w

**Published:** 2021-02-08

**Authors:** Emily R. Bemmer, Kelsie A. Boulton, Emma E. Thomas, Ben Larke, Suncica Lah, Ian B. Hickie, Adam J. Guastella

**Affiliations:** 1 Autism Clinic for Translational Research, Brain and Mind Centre, Child Neurodevelopment and Mental Health Team, Children’s Hospital Westmead Clinical School, Faculty of Medicine and Health, University of Sydney, 100 Mallet Street, Camperdown, NSW 2050 Australia; 2 School of Psychology, University of Sydney, Sydney, 2050 Australia; 3 Child Neurodevelopment and Mental Health Team, Brain and Mind Centre, Children’s Hospital Westmead Clinical School, Faculty of Medicine and Health, University of Sydney, Camperdown, NSW 2050 Australia

**Keywords:** Autism, Social anxiety, Group, Intervention, Cognitive–behavioural therapy, Mental health, Social skills

## Abstract

**Background:**

There is a strong research imperative to investigate effective treatment options for adolescents and adults with autism spectrum disorder (ASD). Elevated social anxiety, difficulties with social functioning and poor mental health have all been identified as core treatment targets for this group. While theoretical models posit a strong bidirectionality between social anxiety and ASD social functioning deficits, few interventions have targeted both domains concurrently. Of the two group interventions previously conducted with adolescents and adults with ASD, significant results have only been observed in either social anxiety or social functioning, and have not generalised to changes in overall mood. The aim of this study was to evaluate the potential benefit, tolerability and acceptability of a group cognitive-behaviour therapy (CBT) intervention in young adults with ASD. Primary treatment outcomes were social anxiety symptoms and social functioning difficulties; secondary outcomes were self-reported mood and overall distress.

**Method:**

Ten groups of participants completed an eight-week, modified group CBT intervention targeting both social anxiety and social functioning, that included social skills training, exposure tasks and behavioural experiment components. Seventy-eight adolescents and young adults with ASD, without intellectual impairment, aged between 16 and 38 (*M* = 22.77; SD = 5.31), were recruited from the community, Headspace centres and the Autism Clinic for Translational Research at the Brain and Mind Centre, University of Sydney. Outcomes (social anxiety, social functioning and mood) were measured pre- and post-intervention via self-report questionnaires (administered either online or through the return of hard-copy booklets), and participants were invited to provide anonymous feedback on the intervention (at the mid-point and end of the intervention).

**Results:**

Participants demonstrated statistically significant improvements on all outcome measures in response to the intervention. Specifically, social anxiety symptoms decreased (*p* < .001), and specific subdomains of social functioning improved post-intervention, particularly in social motivation (*p* = .032) and restricted interests and repetitive behaviours (*p* = .025). Self-reported symptom improvements also generalised to mood (depression, anxiety and stress; *p* < .05). All improvements demonstrated small effect sizes. Participant feedback was positive and indicated strong satisfaction with the program.

**Limitations:**

The absence of a control group and follow-up measures, reliance on self-report instruments as outcome measures and the exclusion of those with intellectual disability represent significant limitations to this study.

**Conclusions:**

These findings indicate that a group CBT intervention appears to be a beneficial intervention for self-reported social anxiety, social functioning and overall mental health in adolescents and young adults with ASD. The stand-alone nature of the intervention combined with positive participant feedback indicates it was well tolerated, has potential clinical utility and warrants further study in a randomised-controlled, follow-up design.

## Background

Autism spectrum disorder (ASD) is known to have significant impacts across the lifespan [1, 2]; however, the research focus and delivery point of interventions to date have typically been restricted to early childhood and school-age years [3]. Given an acknowledged ‘services cliff’ experienced by those with ASD post high-school, there exists a strong research imperative for evidenced-based interventions targeted to young adults, where both the research base and support services are lacking [4, 5].

Adults with ASD are at an increased risk of mental health problems, which have been found to be one of the strongest predictors of disability, reduced quality of life and difficulties in daily functioning [6, 7]. Depression and anxiety have both been identified as core quality of life concerns amongst adolescents and adults with ASD, particularly with the stressful transition into adulthood and increased expectations of social competence [8]. Amongst psychiatric comorbidities, the most common is social anxiety disorder (SAD), which occurs at a much higher rate in adults with ASD (50–70%) [9, 10] compared to the general population (7%) [11]. However, given the 50–70% prevalence estimates are derived from clinical samples, the overall prevalence of SAD in population ASD samples may be lower [7, 9]. SAD is characterised by both persistent, intense fears of negative evaluations in social situations and social avoidance behaviours [11]. The impact and experience of comorbid SAD in adults with ASD have also been captured through qualitative research; with one study participant describing social anxiety in the following way;

As the years pass, I suffer increasing anxiety for lack of even casual acceptance by my species and, conversely, huge spikes of anxiety when someone actually does ‘see’ me. Invisibility has become my comfort zone as well as my prison [12]. p. 481.

A bidirectional link between social anxiety and the key symptoms of ASD, particularly difficulties in social functioning and reciprocal social interactions, has been suggested as a primary contributor to the high co-occurrence of social anxiety in people with ASD [13]. That is, the difficulties with social interaction and communication commonly experienced by those with ASD may lead to an increased prevalence and severity of SAD, and, in turn, this elevated anxiety may further exacerbate preexisting social deficits. Various factors have been identified for maintaining this bidirectional link between social anxiety and social functioning, including physiological arousal, intolerance of uncertainty, social withdrawal, and difficulties expressing and understanding emotion [14, 15]. Further, the peculiarity of special interests, repetitive behaviours and rigidity around routines can isolate people with ASD from their neurotypical peers [16, 17]. This has been found to increase rates of rejection and bullying, thus increasing the vulnerability of people with ASD to negative social experiences [18]. Sensory aversions to certain environments, sounds or lights can induce discomfort and further increase anticipatory anxiety or avoidance behaviours [17, 19]. Consistently, social skills deficits and social anxiety are strongly correlated in both children and adults with ASD [20, 21].

Despite the bidirectional relationship that has been demonstrated to exist between social anxiety and social functioning, anxiety interventions and social skills groups for ASD have predominantly been examined separately. In a recent review of literature, Balderaz (2020) identified six published studies that reported on Group Social Skills Interventions (GSSIs) for adults with ASD. Significant improvements in social functioning were found in four studies, however, across all studies the improvements in social functioning did not generalise to improvements in either social anxiety or general mental health [22]. This stands against the general findings in children and adolescents, with a systematic review of child GSSIs finding that six out of the 10 included studies reported a significant shift in depression and anxiety in pre-post analysis [23]. One study, however, reported contrasting findings, with participants displaying change in social anxiety, but no change in social functioning or general mental health [24]. The lack of change on mood outcomes across GSSIs for adults with ASD is inconsistent with other literature demonstrating the efficacy of group interventions (especially CBT-based) in improving mental health outcomes for adults with SAD [25, 26]. An exception to this was a study that involved adults with ASD (aged 18–29) which reported increases in social functioning and significant small-to-medium treatment effects on mood upon completion of the intervention [27]. The reduction in mood symptoms was suggested to be due to positive social experiences and the support gained from the group intervention, though it remains unclear why this generalisation effect has not been observed in other group studies with adults [27].

For interventions targeting anxiety disorders in ASD (including SAD), there is a small but promising body of emerging research demonstrating the efficacy of modified cognitive–behavioural therapy (CBT) for both children and adults. A recent meta-analysis of 11 studies reported statistically significant improvements on clinician and informant-report measures of anxiety in response to CBT interventions [25]. While this meta-analysis only included two studies with adults, additional randomised-controlled trials have demonstrated the positive effects of CBT-based interventions for transdiagnostic anxiety disorders in children and adults with ASD [28–30]. Wood et al. [31] in their RCT with 167 children found significant improvements in children on their primary anxiety measure, but also found improvements related to social communication. Considering interventions for anxiety in adults with ASD, the majority of literature to date has focussed on those without intellectual disability (ID). Within the broader scope of CBT studies, Weston et al. [25] found of the 24 studies evaluating CBT on various affective and ASD symptoms, the majority involved group interventions (15 in total). Other research has consistently indicated the benefits of group interventions for anxiety disorders generally, both as a cost-effective treatment intervention, but also given the group context provides a natural milieu for both exposure and skills practice [31]. Not surprisingly, these same advantages have been highlighted within GSSIs for people with ASD [32].

At present, there are only two published group intervention studies that target both social skills and social anxiety, and have been modified to suit ASD populations. Both studies involved a combination of group and individual sessions. The first study used the Multimodal Anxiety and Social Skills Intervention (MASSI) and included seven group sessions and up to 13 individual sessions that incorporated parent education and training [33]. In the randomised control trial, 30 teenagers aged 12–17 years were recruited and assigned to either the MASSI intervention or a waitlist control group. Assessments were conducted pre- and post-intervention, with the MASSI showing a large, statistically significant treatment effect on social skills (indexed by the Social Responsiveness Scale; SRS-2) but no statistically significant effect for anxiety [33]. A small sample size was suggested as a potential reason for this. Parent and adolescent measures taken following the program indicated high levels of overall satisfaction with both the group and individual treatment components [33].

The second group intervention study, the ‘Social Skills Intervention’ recruited 18 adult males aged 22–48, for a social skills/social anxiety group following a course of individual CBT [24]. The ‘Social Skills Intervention’ was an 11-week program that covered topics (through a CBT framework) including communication strengths and difficulties, types of relationships, goal setting, conversation skills and emotional awareness of self and others. The model of treatment placed greater emphasis ‘on those interventions derived from cognitive principles’, while behavioural strategies like exposure were used to inform between-session tasks. Assessments were conducted pre- and post-intervention, with a medium effect size on self-reported social anxiety (indexed by the Leibowitz Social Anxiety Scale, Self-Report; LSAS-SR), but changes on measures of low mood, general anxiety and overall social functioning were not significant. Importantly, feedback in the final sessions was reported as positive, with participants indicating tolerability and acceptability of the program [24].

In joint social anxiety and social functioning interventions, the MASSI was found to significantly improve social functioning, while the Social Skills Intervention improved social anxiety but not mood or social functioning [24, 33]. To date, it is not known if equivalent treatment effects across both domains can be achieved without the supplement of individual psychological sessions. The inclusion of individual psychotherapy has been identified as a potential limitation to generalisability and clinical utility in both studies. The purpose of the current study was to determine both the potential benefit and acceptability of an adjusted CBT group intervention for young adults with ASD to reduce social anxiety symptoms and improve social functioning difficulties.

In line with research demonstrating the benefits of CBT programs in improving social anxiety [24] and social functioning [33], and studies in children demonstrating improvements in both domains [30], we hypothesised that the CBT group intervention would result in reductions on both the primary measures of social anxiety and social functioning deficits. Given the theoretical understanding of the bidirectional relationship of social anxiety and social functioning, it was hypothesised that an analysis of predictor variables would support this model, and the predictive influence of demographic factors would provide insight into potential generalisability or limitations of the program. Participant feedback, in line with previous modified CBT studies, was hypothesised to be predominantly positive. Due to the mixed findings in the literature related to the effect of CBT group interventions on general mental health [23, 24, 27], we made no specific hypotheses as to the impact of the intervention on mood or psychological distress.

## Method

This was a pre-post study of a CBT group intervention for social anxiety that had been modified with social skills components specifically for adults with ASD. The ‘Engage Program’ is an intervention that incorporated core CBT components of exposure, cognitive re-structuring, in-session behavioural experiments, and social skills training, and included planning and review of individualised homework tasks [34]. The study was approved by the University of Sydney Human Research Ethics Committee (no. 2015/365). All participants provided written informed consent prior to their inclusion in the study.

### Participants

Participants were recruited through clinical referral or by word-of-mouth, from the community, local Headspace centres (providing mental health services for 12–25-year olds) and from referrals to the Autism Clinic for Translational Research at the Brain and Mind Centre, University of Sydney between January 2016 and March 2020. Inclusion criteria were: participants were help-seeking and either (1) had an ASD diagnosis established within the past 12 months using the Autism Diagnostic Interview-Revised (ADI-R) or Autism Diagnostic Observation Schedule-2 (ADOS-2) (*n* = 9), or (2) were administered the ADOS-2 at study entry (*n* = 79) to confirm they met cut-off scores for autism or autism spectrum disorder. Participants were also required to be at least 16 years of age. Information on current and previous psychological or pharmacological treatment was not collected. Exclusion criteria were: ID (where estimated FSIQ < 70, as assessed by the Wechsler Test of Adult Reading (WTAR)), active psychosis identified during intake assessment, inpatient admission for acute mental health concerns, low English proficiency, substance abuse issues, or significant visual or auditory impairment that would hinder engagement with audio/visual components of the program, or overall treatment engagement. While participants could withdraw before the group commenced (*n* = 1), participants who missed more than three treatment sessions were excluded (*n* = 5). Participants that completed the intervention but did not complete post-questionnaires were included using an intent-to-treat analysis (*n* = 9). The total attrition from the group program was six participants (8%). Reasons for attrition included university timetable clashes, relocation to another state and low motivation (referral from parent, but no reported motivation to attend by the individual).

**Fig. 1:**
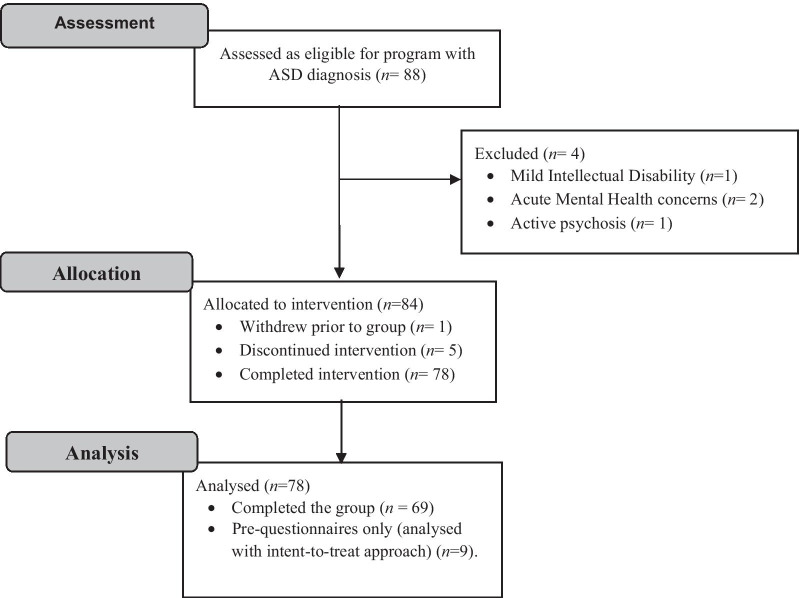
CONSORT diagram for study participants

Eighty-eight participants were initially assessed as eligible for study, as shown on the CONSORT diagram (Fig. 1). Four participants met exclusion criteria, and six participants withdrew or discontinued the intervention. Seventy-eight participants were in the final sample (47 males, 30 females, 1 non-binary) and were between 16 and 38 years of age (*M* = 22.77, SD = 5.31). In total, 10 intervention groups were run at the University of Sydney Brain and Mind Centre.

### Intervention program

#### Development

The CBT program used in the current study was developed from established social anxiety treatment programs for adults [35, 36]. The adaptations considered needs of adults with SAD and comorbid ASD who have difficulty implementing typical cognitive interventions due to limited introspection and a poorer understanding of social rules and norms [37]. To make the anxiety-based interventions more effective for adults with ASD, the current intervention included structured frameworks for teaching of social skills, such as entering and maintaining conversations, and managing disagreements. In addition, cognitive work (such as identifying and challenging negative beliefs) was simplified and used to support the behavioural components (role plays, exposure tasks and out-of-session practice tasks) that formed the core interventions in the program. Behavioural interventions were integrated *within* treatment sessions, and as a focus of weekly homework to facilitate engagement and promote positive treatment outcomes. Such alterations have been strongly recommended for interventions targeting anxiety in ASD, both in child [38, 39] and adult populations [40, 41].

#### Procedure

Before commencing the eight-week group intervention, participants completed a battery of self-report measures assessing social functioning, symptom severity and mood. These measures were also completed upon completion of the intervention. Measures of social anxiety and social functioning were included as primary outcome measures, and measures of mood were included as secondary outcome measures. These measures were selected on the basis of their reliability in tracking outcomes relevant to the aims of the study, and from their previous use in assessing symptoms and treatment responsiveness in adults with ASD [6, 17, 42].

The current modified-CBT program was delivered to groups of six to eight participants over eight consecutive weekly sessions that took approximately two and a half hours each. Two clinicians facilitated groups. By the end of the first session, participants were required to self-nominate a social support person to help practice skills, increase compliance with homework tasks and to increase the ability to generalise the application of CBT strategies to different social contexts. Unlike other interventions, the social support person was not required to be a parent, and could be a partner, housemate or close friend to provide support. Across the 78 participants, only five were unable to find a suitable support person.

Across the eight sessions, a consistent structure was maintained. Groups commenced with a short anxiety-reduction exercise (either breathing or body scan) to assist with focus and engagement for the session. This was then followed by an extended homework review (or a brief role-play task for those without completed homework to discuss). Homework completion was not formally monitored, but informally, through allocation of alternative tasks when uncompleted. Homework tasks included making phone calls to other group members, completion of thought/anxiety monitoring, and completing planned exposure or social engagement activities. Participants then completed a block of core content, that covered CBT for social anxiety and social skills training. Table 1 provides an overview of the core content areas of the modified-CBT program.

**Table 1:** Outline of core CBT components across the eight-week program

Session	Approx. time	Core components
1	5 min 55 min 60 min 30 min	Paced breathing exercise Orientation to CBT skills group Social skills training Homework allocation and Café time (skills practice/exposure)
2	5 min 45 min 70 min 30 min	Paced breathing exercise Homework review Social anxiety psychoeducation Avoidance and exposure Homework allocation and Café time (skills practice/exposure)
3	5 min 45 min 60 min 30 min	Paced breathing exercise Homework review Social skills training Homework allocation and Café time (skills practice/exposure)
4	5 min 30 min 15 min 70 min 30 min	Paced breathing exercise Homework review Psychoeducation: Negative thinking patterns and behavioural experiments In session behavioural experiment Homework allocation and Café time (skills practice/exposure)
5	5 min 45 min 20 min 35 min 15 min 30 min	Body scan/grounding exercise* Homework review Behavioural experiment Social skills training Psychoeducation: Selective attention Homework allocation and Café time (skills practice/exposure)
6	5 min 45 min 20 min 50 min 30 min	Body scan/grounding exercise Homework review Anxiety surfing Social skills training Homework allocation and Café time (skills practice/exposure)
7	5 min 45 min 20 min 50 min 30 min	Body scan/grounding exercise Homework review Behavioural activation Social skills training Homework allocation and Café time (skills practice/exposure)
8	5 min 45 min 30 min 25 min 15 min 30 min	Body scan/grounding exercise Homework review Behavioural experiment Relapse and response prevention Group wrap up Café time (skills practice/exposure)

^*^Introductory exercise changed from breathing to grounding at week 5 to enable participants to gain mastery of two techniques

Following the core content, relevant homework was explained and allocated. The final 30 min of the group program was ‘café time’, where participants practiced skills learnt in session in a kitchen area, as a closer approximation to ‘real life’. This café time was also used by facilitators for individual follow-up, to plan appropriate homework tasks and address participant’s specific fears or negative thinking patterns. Participants’ nominated support person was emailed each week with a copy of the session slides and additional explanatory materials that provided suggestions to generalise skill development throughout the week.

#### Facilitators

Two members of clinical staff (clinical psychology, E.B; B.L; M.C; clinical social work, E.T) facilitated group sessions. All staff had experience and clinical training facilitating groups with adolescents and adults with ASD. Each group session was followed by a 60 min debrief between the facilitators involved, which included formulation and review of specific goals for individual participants.

### Measures

*Autism Diagnostic Observation Schedule-2 (ADOS-2) *[43]. The ADOS-2 assesses ASD symptomatology in children and adults. The ADOS-2 consists of a semi-structured observational assessment, with scores generated across three domains; social interaction, communication and imaginative use of materials. Module four, designed for verbally fluent older adolescents and adults, was used in the current study. Higher scores for each domain indicate increased symptom severity.

*Wechsler Test of Adult Reading (WTAR) *[44]*.* The WTAR is a neuropsychological assessment tool that provides an estimate of Full-Scale Intelligence Quotient (FSIQ; *M* = 100, *SD* = 15) based on participants’ age-normed ability to read aloud 50 irregular words of increasing difficulty.

#### Primary outcome measures

*Liebowitz Social Anxiety Scale—Self-Report (LSAS-SR) *[45]*.* The LSAS-SR is a 24-item measure assessing anxiety and avoidance of social situations. Two subscale scores (avoidance and fear) and a total score are derived from the measure, with higher scores indicate of greater symptom severity. Social anxiety difficulties are indicated within the following ranges; 50–64: moderate social phobia, 65–79: marked social phobia, 80–94: severe social phobia, ≥ 95: very severe social phobia. The LSAS-SR is one of the most commonly used measures of social anxiety in adult ASD populations [24, 46]. The scale had high internal consistency, as determined by a Cronbach's alpha of 0.96.

*Social Responsiveness Scale-2—Adult Self-Report (SRS-2) *[47]*.* The SRS-2 is a 65-item rating scale that measures social skill functioning and ASD symptoms in adults. Five subscale scores, measuring social ‘Awareness’, ‘Cognition’, ‘Communication’, ‘Motivation’ and ‘Restricted Interests and Repetitive Behaviours’ (RRB), as well as a total score are derived from the measure. Both the RRB and a combined ‘Social Communication and Interaction’ subscale are compatible with DSM-5 criteria for ASD. Raw scores on the SRS-2 are converted to *T* scores which are indicative of social functioning difficulties within the following ranges; ≤ 59: normal, 60–65: mild difficulties, 66–75: moderate difficulties, ≥ 76: severe. The scale had high internal consistency, as determined by a Cronbach's alpha of 0.94.

#### Secondary outcome measures

*Depression Anxiety Stress Scales (DASS-21) *[48]. The DASS-21 is a self-report measure of depression, anxiety and stress, and assesses symptom severity over the past week, and has recently been validated for use in ASD populations [49]. Higher scores correspond to increased symptom severity. Severity ranges (after doubling the raw scores) are indicated for depression as; 10–13: mild, 14–20: moderate; 21–27: severe, ≥ 28: extremely severe. For anxiety, ranges are: 8–9: mild, 10–14: moderate, 15–19: severe, ≥ 20: extremely severe. For stress, ranges are; 15–18: mild, 19–25: moderate, 26–33: severe, ≥ 34: extremely severe. The scale had high internal consistency, as determined by a Cronbach's alpha of 0.95.

*Kessler Psychological Distress Scale (K10) *[50]*.* The K10 is a well-validated 10-item rating scale commonly used to measure psychological distress over the past four weeks. Higher scores correspond to greater self-reported distress. Scores ranges on the k10 indicate level of psychological distress as; 20–24; mild, 25–29; moderate; 30–50; severe level of disorder. It has been used in similar studies to measure overall symptoms of distress, rather than disorder-specific (anxiety/depression) symptoms in adults with ASD [6]. The scale had high internal consistency, as determined by a Cronbach's alpha of 0.92.

*Social Interaction Anxiety Scale (SIAS) and Social Phobia Scale (SPS) *[51]*.* The SIAS and SPS are partner measures used to assess social anxiety and have previously been used to measure SAD levels in ASD populations [10]. Both measures have also been recently validated for use in ASD populations [52]. The SIAS requires participants to rate 20 items about anxiety related to initiating and maintain conversations, while the SPS requires ratings on 20 items related to fears of being observed or evaluated in daily activities (public speaking, eating etc.) [53]. Clinical cut-off scores to indicate the suggested presence of SAD for the SIAS and SPS are 34 and 24, respectively. Higher scores on each measure indicate greater symptom severity. The scales had high internal consistency, as determined by a Cronbach's alpha of 0.86 (SIAS) and 0.94 (SPS).

*Tolerability measures.* Participants were also invited to complete a survey at the mid-point of treatment, assessing expectations of, and engagement with the intervention, as well as potential barriers. This questionnaire consisted of six free response, and two Likert-scale questions (refer to “Appendix A”). Upon completion of the intervention, participants were offered the opportunity for a one-on-one interview with a group facilitator, and were invited to provide written feedback on their experience of the intervention.

### Statistical analysis

An a priori power analysis was conducted using G*Power 3 [54] to test the differences between two paired-sample group means using a two-tailed test, a small-medium effect size (*d* = 0.40) and an alpha of 0.05. Result showed that a total sample of 52 pairs was required to achieve a power of 0.80.

All analyses were two-tailed, and alpha was set at 0.05. Statistical computations were performed using the Statistical Program for Social Science (SPSS), version 26. Data were inspected visually for normality, and using skewness, kurtosis values and Shapiro–Wilk’s test of equality of variance. All data met normality assumptions. Primary analyses were undertaken on an intention-to-treat basis, including all eligible participants. Multiple imputation was used to handle the missing data. The multiple imputations were conducted with the Markov Chain Monte Carlo (MCMC) method with 10 iterations using predictive mean matching for missing values. Paired-samples t tests were applied to compare pre-treatment to post-treatment scores on self-report questionnaires.

Multiple regression analysis examined the predictive value of demographics variables and the alternate primary outcome measures (age, gender, IQ estimate, ADOS-2 total score and either LSAS-SR or SRS-2 total change scores) on social anxiety or social skills change (indexed by the LSAS-SR or SRS-2 total change score). Cohen’s *d* was calculated to determine treatment effect sizes, using the accepted cut-offs of 0.2 (small), 0.5 (medium) and 0.8 (large) [55].

## Results

### Pre- and post-completers—missing analysis

Considering participants who completed questionnaires both pre- and post-intervention (*N* = 69), there were 242 missing data points out of 2932 possible (92.38% completion rate). Little’s Missing Completely at Random test indicated randomness in the missing data, *χ*^2^ (389) = 314.29, *p* = 0.998.

### Demographics

Descriptive statistics were calculated for age, WTAR estimated IQ, and gender for the sample (see Table 2). Independent-samples t tests showed no statistically significant differences in IQ estimate (*t*(75) = 1.08, *p* = 0.278), ADOS total scores (*t*(75) = 0.464, *p* = 0.643) or age between males and females (*t*(75) = 0.103, *p* = 0.918). Likewise, there was no statistically significant difference in the number of males and females in the complete sample (*χ*^2^ (1, *N* = 78) = 3.753, *p* = 0.053). As illustrated in Table 2, severity scores on the ADOS-2 ranged from mild to severe, consistent with prior studies using a similar sample [6, 56]. As also shown in Table 2, post-questionnaire non-completers were found to have significantly lower self-reported social anxiety on all subscales of the LSAS (*p* < 0.05). All other demographic and baseline measure differences were not significant (*p* > 0.05).

**Table 2:** Baseline demographics and mean questionnaire results overall, between completers and non-completers

Variable	Complete sample (*n* = 78)	Intervention completers (*n* = 69)		Post-questionnaire non-completers (*n* = 9)		Significance test
**Demographics**	***M (SD)***	***M (SD)***	**Range**	***M (SD)***	**Range**	***p value***
Age	22.77 (5.31)	22.30 (4.7)	16–33	26.33 (8.17)	18–38	.147
WTAR (FSIQ estimate)	106.32 (11.66)	106.23 (11.89)	73–129	107 (10.25)	83–119	.854
ADOS-2 total score	10.16 (2.91)	10.16 (2.89)	7–17	10.11 (3.22)	7–18	.956
**Gender**	***N (%)***	***N (%)***		***N (%)***		
Male	47	39		8		
Female	30	29		1		
Other	1	1		0		
**Social anxiety**	***M (SD)***	***M (SD)***	**Range**	***M (SD)***	**Range**	***p value***
LSAS-SR total	79.78 (27.36)	82.20 (23.93)	22–135	61.22 (22.1)	31–102	.013
LSAS-SR fear	42.17 (14.81)	43.37 (12.49)	12–69	33 (14.78)	11–57	.022
LSAS-SR avoidance	37.62 (13.74)	38.84 (12.35)	10–66	28.22 (8.21)	20–45	.001
SIAS total	45.51 (14.09)	46.01 (14.61)	17–80	41.78 (13.86)	18–57	.412
SPS total	31.41 (19.06)	32.45 (18.79)	2–70	23.78 (17.73)	2–49	.191
**Social functioning**	***M (SD)***	***M (SD)***	**Range**	***M (SD)***	**Range**	***p value***
SRS total	70.62 (9.7)	71.05 (11.23)	36–90	67.55 (9.33)	52–83	.374
SRS-awareness	60.41 (9.3)	60.42 (10.11)	32–81	60.44 (6.33)	49–69	.993
SRS-cognition	65.5 (10.47)	65.69 (11.42)	37–88	64.11 (7.83)	56–79	.688
SRS-communication	70.54 (9.75)	70.78 (11.52)	37–87	68.77 (10.8)	54–85	.622
SRS-motivation	70 (9.26)	70.49 (10.77)	37–89	66.44 (11.09)	51–79	.292
SRS-SCI	69.23 (9.43)	69.43 (11.02)	35–89	67.77 (9.16)	54–83	.668
SRS-RRB	71.14 (11.19)	71.93 (12.77)	40–90	65.33 (11.82)	45–82	.143
**Mood and distress**	***M (SD)***	***M (SD)***	**Range**	***M (SD)***	**Range**	***p value***
DASS-21-depression	20.29 (12.06)	20.81 (12.9)	0–42	16.44 (8.05)	0–24	.325
DASS-21-anxiety	16.05 (11.15)	16.51 (11.35)	0–42	12.67 (9.8)	0–30	.334
DASS-21-stress	22.87 (10.86)	23.37 (10.89)	0–42	19.11 (11.83)	0–42	.275
K10	27.69 (9.76)	28.05 (9.59)	0–50	25 (10.44)	10–47	.375

WTAR (FSIQ estimate) = Wechsler’s Test of Adult Reading estimate of Full-Scale Intelligence Quotient; ADOS-2 = Autism Diagnostic Observation Schedule, 2nd Edition; LSAS-SR = Leibowitz Social Anxiety Scale-Self Report; SIAS = Social Interaction Anxiety Scale; SPS = Social Phobia Scale; SRS-2 = Social Responsiveness Scale 2nd Edition t scores; SRS-SCI = Social Responsiveness Scale—Social Communication and Interaction; SRS-RRB = Social Responsiveness Scale—Restricted Interests and Repetitive Behaviour; DASS-21 = Depression, Anxiety, Stress Scale—21 item; K10 = Kessler Psychological Distress Scale

### Primary outcomes

#### Social anxiety

Table 3 shows the results for the primary outcome measures of Social Anxiety (as indexed by the LSAS-SR, SIAS and SPS) for the complete sample. With the exception of the SPS (*p* = 0.056), comparisons of scores pre- and post-intervention indicated statistically significant reductions across all social anxiety measures and subscales (LSAS-SR total, LSAS-SR fear, LSAS-SR avoidance and SIAS) (*p* < 0.005) with small- effect sizes (Cohen’s *d* ranging from 0.28 to 0.35).

**Table 3:** Baseline and post-intervention scores obtained on measures of social anxiety and social functioning, statistical analysis and effect sizes on primary outcome measures

Variable	Pre-group mean (SD)	Post-group mean (SD)	Mean change (SD)	*t (df)*	*p*	Cohen’s d
LSAS-SR (total)	79.78 (27.36)	70.17 (31.04)	9.61 (20.41)	*t*(77) = 4.05	< *.*001	0.33
LSAS-SR (fear)	42.17 (14.81)	36.87 (15.77)	5.3 (11.1)	*t*(77) = 4.03	< *.*001	0.35
LSAS-SR (avoidance)	37.62 (13.74)	33.31 (16.41)	4.31 (10.83)	*t*(77) = 3.62	.003	0.28
SIAS-total	45.51 (14.09)	40.86 (14.64)	4.6 (11.71)	*t*(75) = 3.31	*.*002	0.32
SPS-total	31.41(19.06)	28.26 (17.46)	3.15 (13.53)	*t*(74) = 1.76	.056	0.17
SRS-total	70.62 (9.7)	68.95 (9.3)	1.67 (8.75)	*t*(73) = 1.63	*.*103	0.18
SRS-awareness	60.41 (9.3)	62.33 (9.1)	− 1.91 (11.08)	*t*(73) = − 1.46	.145	− 0.21
SRS-cognition	65.5 (10.47)	64.96 (9.48)	0.54 (11.28)	*t*(73) = 0.41	*.*683	0.05
SRS-communication	70.54 (9.75)	69.02 (10.02)	1.52 (9.60)	*t*(73) = 1.34	.181	0.15
SRS-motivation	70 (9.26)	67.15 (8.38)	2.85 (9.23)	*t*(73) = 2.66	.008	0.32
SRS-SCI	69.23 (9.43)	67.78 (8.97)	1.44 (10.72)	*t*(73) = 1.09	.278	0.16
SRS-RRB	71.14 (11.19)	68.31 (11.73)	2.83 (9.48)	*t*(73) = 2.5	*.*013	0.25

LSAS-SR = Leibowitz Social Anxiety Scale-Self Report; SIAS = Social Interaction Anxiety Scale; SPS = Social Phobia Scale; SRS-2 = Social Responsiveness Scale 2nd Edition; SRS-SCI = Social Responsiveness Scale-Social Communication and Interaction; SRS-RRB = Social Responsiveness Scale-Restricted Interests and Repetitive Behaviour

#### Social functioning

Results for Social Functioning (as indexed by the SRS-2) are shown in Table 3 below. While the change in overall functioning (SRS-2 total score) was not significant, there was a significant decrease on the ‘Social Motivation’ domain from pre- to post-intervention (*p* = 0.008, *d* = 0.35). This indicates fewer difficulties in social engagement, and increased social motivation following the intervention. There was also a statistically significant decrease in the ‘Restricted and Repetitive Behaviours’ domain (*p* = 0.013, *d* = 0.25).

### Secondary measures

#### Mood and distress

As illustrated in Table 4, participants also showed statistically significant improvement in mood, anxiety and stress (as indexed by the DASS-21) with small effect sizes (*p* < 0.05, with Cohen’s *d* ranging from 0.28 to 0.35). The small effect size for reduction in psychological distress (K10) was not significant (*p* = 0.121)*.*

**Table 4:** Baseline and post-intervention raw scores, statistical analysis and effect sizes on secondary outcome measures

Variable	Pre-group Mean (SD)	Post-group Mean (SD)	Mean change (SD)	*t (df)*	*p*	Cohen’s d
DASS-21-depression	20.29 (12.06)	17.03 (11.01)	3.26 (11.72)	*t*(75) = 2.28	*.*024	0.28
DASS-21-anxiety	16.05 (11.15)	12.74 (9.08)	3.32 (9.78)	*t*(75) = 2.76	*.*006	0.33
DASS-21-stress	22.87 (10.86)	19.18 (10.17)	3.68 (9.29)	*t* (75) = 3.19	*.*002	0.35
K10	27.69 (9.76)	25.81 (8.55)	1.88 (8.73)	*t*(76) = 1.59	.121	0.2

DASS-21 = Depression, Anxiety, Stress Scale-21 item; K10 = Kessler Psychological Distress Scale

Multiple regression analyses were calculated to predict change on the primary outcome variables (LSAS-SR and SRS-2) based on demographics variables (age, gender, WTAR IQ estimate and ADOS-2 total scores) and either baseline social anxiety (LSAS-SR) or social functioning (SRS-2). As shown in Table 5, demographics and pre-intervention social functioning did not significantly predict changes on the LSAS-SR total score (*F*(5, 67) = 1.02, *p* = 0.421, *R*^2^ = 0.07). However, for the SRS-2 change scores, a significant regression equation was found (*F*(5, 67) = 3.112, *p* = 0.015, *R*^2^ = 0.189). Of the predictors, only the baseline social anxiety score (LSAS-SR total) was a significant predictor of social functioning change post-intervention, such that higher social anxiety symptoms pre-intervention predicted greater improvement in social functioning (ϐ = 0.26, *t*(67) = 2.18, *p* = 0.030). All other predictors were not significant (*p* > 0.05).

**Table 5:** Summary of regression analyses of primary outcome measures

Variable	*B*	SE_B_	ϐ	*p*
LSAS-SR—total change score				
Intercept	− 34.73	35.45		.328
Age	− 0.11	0.48	− 0.03	.82
Gender	8.26	5.34	0.2	.112
WTAR (FSIQ estimate)	0.24	0.23	0.13	.311
ADOS-2 total scores	-0.45	0.88	− 0.06	.612
SRS-2 pre-intervention	0.20	0.25	0.11	.424
SRS-2—total change score				
Intercept	6.52	11.34		.565
Age	0.17	0.19	0.01	.925
Gender	3.12	2.1	0.13	.136
WTAR (FSIQ estimate)	− 0.12	0.84	− 0.16	.164
ADOS-2 total scores	− 0.48	0.34	− 0.16	.159
LSAS-SR pre-intervention	0.1	0.04	0.26	.030

LSAS-SR = Liebowitz Social Anxiety Scale, Self-Report; SRS-2 = Social Responsiveness Scale, 2nd edition; WTAR (IQ estimate) = Wechsler’s Test of Adult Reading estimate of Full-Scale Intelligence Quotient; ADOS-2 = Autism Diagnostic Observation Schedule, 2nd edition; LSAS = Liebowitz Social Anxiety Total Score Pre-Group; Scale *B* = unstandardised regression coefficient, SE_B_ = standard error of the coefficient; ϐ = standardised coefficient

### Tolerability measures

While all participants were invited to provide feedback on the intervention, 28 participants (47%) completed the form. For the item, ‘I am enjoying the group’ 96% of participants indicated they ‘agree’ or ‘strongly agree’. No participants indicated ‘disagree’ or ‘strongly disagree’ on this item. Prior knowledge about anxiety varied across participants. On open-ended responses, participants indicated that making friends, being able to talk and ask questions without judgement, feeling understood by others and having practical help and support were working well within the group. Participants indicated that making phone calls, feeling like their anxiety was hindering their learning, and finding the groups either too long (three participants) or too short (two participants) were difficulties with the group. Participants generally reported positive engagement with other group members, though some reported difficulties with a specific group member (being too talkative or disruptive). Participants were also invited to provide written feedback on their experience of the intervention; with selected responses included in Table 6.

**Table 6:** Qualitative reports provided by participants following the group program

Participant 1	“I’ve always hated phone calls, they’re probably the one thing that make me most anxious. This week I was able to make a phone call to one of our other group members. I didn’t want to do it, but we ended up having a great conversation… and we talked for 50 min!”
Participant 2	“It was also really nice to be in a group where everyone shares similar problems that other people can take for granted. I didn’t feel the need to ‘conform’ to neuro-typical behaviour, which took a lot of pressure off. Everyone actually understands, which is really rare. I have since been able to stay in contact with everyone post-group, which again is nice because I still have connections with people who ‘get it”
Participant 3	“…for the longest time I always felt like there were these 'unspoken rules' of society that everyone else had been taught except me, and no matter how hard I tried to find out what I was missing, nothing seemed to lay out the bare-bones foundations of how people interact, become friends, and stay friends in a way I could understand”
Participant 4	“It may not sound like much, but I’ve never been able to order food for myself at a shop. I feel I don’t know what to do and I get so anxious I’m worried they won’t even hear me. But I was able to buy myself lunch this week—it’s something I’ve never done and makes me feel like a proper adult now”
Participant 5	“It has been wonderful being in the group and it's unfortunate that it's the last week. I hope to put all your teachings into practice and not give up. I'm thankful for the guidance and it's given me hope to keep persisting, even though there are roadblocks I need to see the achievements so it can help me keep going”

## Discussion

Our study is the first to show statistically significant improvements across both social anxiety symptoms and social functioning domains in young adults with ASD upon completion of a group-delivered CBT intervention specifically adapted for use with this clinical population. Specifically, improvements in social anxiety were observed across both core symptoms, reduction in fear and avoidance of social situations. Improvements in social functioning included increased social motivation and a reduction of restricted and repetitive behaviours, but no significant changes in social awareness, social cognition, social communication, responsiveness or overall social functioning. Significant improvements were also evident for secondary outcomes, including overall mental health (depression, anxiety, stress). While improvements on primary outcomes were not predicted by demographics variables, it was found that higher baseline social anxiety was predictive of increased improvement on social functioning. The inverse result was not found. Participants also reported a strong satisfaction with the program. These results provide promising preliminary evidence for this modified group CBT intervention for improving self-reported social anxiety, social motivation and overall mental well-being in young adults with ASD.

Following this eight-week stand-alone group intervention, participants reported significantly lower social anxiety levels across the subdomains of avoidance, fear and social interaction anxiety. These observed reductions in social anxiety are consistent with prior research indicating the efficacy of CBT in the treatment of SAD in ASD [25]. The observed small treatment effect sizes found are comparable to that reported by Spain et al. [24]. Acknowledging the high prevalence of anxiety in adult ASD populations (50–70%), the necessity to successfully reduce anxiety is critical. The reduction observed may be linked to the inclusion of components that have demonstrated efficacy in both ASD and non-ASD interventions; exposure, behavioural experiment and challenging negative thoughts [31, 35]. While CBT for SAD has typically demonstrated large effect sizes for those without ASD [26], it is important to acknowledge that given the complex interplay of social anxiety and social deficits in ASD, small treatment effects can be clinically meaningful, and lower self-report scores have often been accompanied by higher informant (usually parent) reports and measures [57].

Unlike prior intervention studies targeting both social anxiety and social functioning [24, 33], the current study was the first to show statistically significant improvements across both social anxiety and domains of social functioning. The small effect-size found on social motivation and RRB as a result of group skills intervention is also consistent with prior studies [22]. These results must be interpreted with caution as the overlap or bidirectionality between social anxiety and social functioning has not been fully explored, and the shift in self-reported social functioning may not be an independent construct [20]. However, it is interesting that an RCT targeting romantic relationships skills for adults with ASD [58] reported similar improvements within the same domains (‘Social Motivation’, and ‘Restricted and Repetitive Behaviours’). As was argued within that study, the focus on communication skills may have contributed towards these reductions. It is not yet known why improvement was observed for ‘Social Motivation’ and not similar domains (such as ‘Communication’ or ‘Awareness’). However, the lack of change in ‘Social Awareness’ suggests some protection against an emerging concern in the literature that social functioning changes in GSSIs are moderated by *awareness* of skills rather than actually *enacting* them [59]. The requirement of at least one (if not multiple) opportunities for skills practice for each skill, and the emphasis placed on accountability for completion of out-of-session tasks also aimed to protect against this concern.

In terms of individual differences and baseline predictor variables, our findings indicate that the level of improvement in social functioning was significantly predicted by baseline social anxiety. That is, individuals with higher social anxiety symptoms at the beginning of the intervention demonstrated greater improvements in social functioning at the completion of the intervention, as compared to those participants with lower baseline social anxiety symptoms. Interestingly, the inverse result was not found, with baseline social functioning not significantly predicting improvements in social anxiety symptoms. This may support the hypothesis that heightened social anxiety hinders the use of social skills that are already present, and so targeting social anxiety via group interventions facilitates additional capacity to apply social skills [17]**.** It should also be noted that none of the demographic variables were significant predictors of social anxiety or social functioning change (*p* > 0.05). For age, the groups included people aged between 16 and 38 based on the needs of the youth clinical services. These needs and responses for participants at different ages, and with differing demographic factors may require further evaluation. While age did not predict change in this study, ad hoc feedback from the therapists suggested those still attending school often had less insight into their social skills needs and perhaps this was due to the social structures that are often provided in schools. These ad hoc reports require evaluation in future studies.

Overall, this analysis of outcome predictors provides support for a unidirectional influence of social anxiety on social functioning in ASD [13] and provides preliminary support for the generalisability of the current program for further use in adult ASD populations. Although the current analysis only supports a unidirectional model, future research evaluating the validity of a bidirectional model of social anxiety and social functioning as a primary aim can utilise measures that may more effectively investigate the moderating relationships between them.

In addition to the primary effects, self-reported depression, anxiety, stress and psychological distress were found to improve following the intervention. The small effect sizes found align with those also reported on the DASS by the GSSI in Leung et al. [28], though, as there was no control group, change may be attributable to factors other than the intervention (such as from the effort and routine change of attending a program). However, as was suggested for their study, behavioural activation components, an awareness of functional improvement and support from peers may have contributed to the improvements in mood. Unlike other studies, the incorporation of the ‘café time’ each week created a specific opportunity for both skills practice and building group rapport. The significance of these generalised improvements stems from prior research indicating that mental health outcomes may be one of the strongest predictors of the level of disability in ASD populations [6]. That is, reduced mental health may be one of the largest contributors to functional impairments and disability burden for adults with ASD. The generalisation of improvement to overall mental health in the current study supports theoretical models that implicate social dysfunction (and associated social anxiety) in the increased prevalence and severity of depressive and other axis I disorders in ASD populations [14].

Feedback regarding participant satisfaction with the group program was positive. The voluntary surveys and exit-interviews indicated enjoyment of the group, application of skills outside the group context and a tangible awareness of the impact of the program in participants’ daily lives. The attrition rate of the group was comparable to that reported in other trials with adult ASD populations [24]. Homework completion was strongly reinforced throughout the program, with in-session behavioural tasks allocated where homework was not completed, whereby skills practice was ensured as much as possible. However, homework compliance was not formally measured in the study.

### Strengths and clinical utility

While most research conducted in adults with ASD involves case studies or small sample sizes, the 78 adults included in the current study make it one of the largest samples for adult ASD mental health treatment to date. Further, the study stands as one of the few with representation of females with ASD, with the predictor analysis finding that gender was not a predictor for treatment efficacy. As none of the demographic factors (sex, age, IQ, ADOS-2 severity) were statistically significant predictors of treatment outcomes, there were no clearly identified barriers to treatment in the current study (e.g. younger age, reduced improvement for one gender) which provides a positive base for further research and transfer to broader clinical contexts. Within clinical practice, the necessity for resource-efficient and efficacious treatment options is critical, particularly given the prevalence and scope of psychiatric comorbidities within ASD populations [9]. The short-term group modality of the current treatment stands as a resource-effective means of providing intervention, with reduced therapist requirements and opportunity for peer learning and support. In addition, group interventions are likely critical for social anxiety and social functioning as the group context provides both exposure to feared situations and ‘real-world’ rehearsal of social skills. Finally, unlike some studies in the field that recruited from specialist ASD services, participants in the current study were recruited from a variety of sources, including the community, which increases treatment portability and reduces potential barriers to treatment.

### Limitations and future directions

This study should also be considered in light of its limitations. The lack of a control group means that pre-post treatment gains cannot be directly attributed to the group intervention alone. Further, the maintenance of treatment effects over time was not investigated. However, our results warrant further investigation by way of a randomised, controlled study design, with re-administration of measures at least 3-months following intervention to assess the maintenance of treatment effects. While this is the largest study to date in this area, future studies with larger sample sizes could reveal predictors of treatment response. A further limitation, which is common to ASD studies, was the reliance on self-report measures developed for use in typically developing populations. While psychometric validation of these measures has been investigated [49, 52] the impact of limited introspection and alexithymia in ASD populations remains a concern within self-report outcomes [25, 60]. In addition, the SRS-2 has been shown to be strongly influenced by co-occurring psychiatric symptoms (such as anxiety) in children and adolescents [61] and so SRS-2 results may be influenced by the reductions observed on social anxiety measures. However, anecdotally, support persons reported to facilitators that they observed functional changes including increased ability to make and maintain conversations (in person and over the phone), and increased social engagement with peers. This reliance on self-report measures meant non-specific factors that may influence responses (e.g. social desirability) could not be controlled. Future research could address these concerns through the inclusion of clinician or informant-report measures and a secondary ASD symptom measure to ensure the validity of outcomes found. It is also important to acknowledge that participants meeting criteria for ID were excluded, and further research is required to establish efficacious treatments, or further adaptations and preparatory work required for the significant proportion of adults with ASD that have comorbid ID. Additional demographic factors, including employment, education, relationship status, as well as extent of current or previous psychological and pharmacological treatment would further inform future research directions. Finally, future studies should conduct structured intervention analysis to determine components of the program that led to positive outcomes, as well as comprehensive qualitative investigations, including thematic analysis, should be conducted to further understand both participant outcomes and experiences of the intervention.

There has been some debate about the potential of Social skills interventions to improve functioning and mental health, or, alternatively, to increase ‘camouflaging’ which is also linked to poorer mental health [62]. Our clinical view is that supports provided in this program provide options for individuals to maintain their own sense of control and agency in social situations. In contrast, we are careful to ensure that individuals do not feel obligated, a loss of control or feel pushed into behaving a certain way. Further evaluation of the environmental impact of the education and support role played by the support person is also required.

## Conclusions

In conclusion, this study found that young adults with ASD who participated in our eight-week modified CBT program reported significant improvements in self-reported social anxiety, social motivation, depression, anxiety and stress. While limitations of the study mean that improvements found cannot be directly attributable to the intervention alone, these findings build upon the small, but promising research base of interventions for adults with ASD and reinforce the necessity for further research in this area. The reflection of one participant captures the importance and necessity for accessible, effective treatments for adults with ASD;*“…now that I know where I stand with people…I’m much more confident in my ability to navigate social interactions and life in general, and I’m much less scared of pursuing the things I want to be doing in life”.* Overall, these results provide promising preliminary evidence supportive of a combined social anxiety and social skills CBT group for young adults with ASD, with strong participant acceptability and potential clinical utility.

## Data Availability

Dataset can be made available on request.

## Appendix A

### Mid-point participant survey

The following survey was administered following week four of the intervention;

- What expectations did you have starting the group?
- Was there anything you were worried about when starting?
- Rate your level of understanding of anxiety before starting this group *(5-point Likert scale)*
- What is going well in the group?
- “I am enjoying the group” (Rate agreement-*5-point Likert scale)*
- Is there anything you are finding difficult?
- What could be improved?
- Is there anything else you would like to feed back to the facilitators at this point?
